# Nontuberculous mycobacteria isolated from specimens of pulmonary tuberculosis suspects, Northern Tunisia: 2002–2016

**DOI:** 10.1186/s12879-019-4441-1

**Published:** 2019-09-18

**Authors:** Reem Gharbi, Besma Mhenni, Saloua Ben Fraj, Helmi Mardassi

**Affiliations:** Unit of Typing & Genetics of Mycobacteria, Laboratory of Molecular Microbiology, Vaccinology, and Biotechnology Development, Institut Pasteur de Tunis, Université de Tunis El Manar, 13 Place Pasteur, BP74, 1002 Tunis, Tunisia

**Keywords:** Nontuberculous mycobacteria, Pulmonary NTM, Pulmonary disease; molecular identification, Phylogeny, Tunisia

## Abstract

**Background:**

Reports on the worldwide ascending trend of pulmonary nontuberculous mycobacteria (NTM) isolation rates and their effective role in respiratory tract infections are compelling. However, as yet, there are no such data relating to Tunisia.

**Methods:**

Here we carried out a retrospective review of mycobacterial cultures originating from Northern Tunisia, which have been processed in the laboratory of mycobacteria of the Institut Pasteur de Tunis, during the time period 2002–2016. All pulmonary NTM (PNTM) isolates available for culture were characterized phenotypically and their taxonomic status was further established based on polymorphisms in *rpoB*, 16S rRNA, *hsp65,* and *sodA* DNA gene sequences.

**Results:**

Of the 10,466 specimens collected from HIV-negative Tunisian patients with presumptive clinical pulmonary TB, 60 (0.6%) yielded PNTM isolates. An overall annual PNTM isolation prevalence of 0.2/100,000 was estimated. As far as could be ascertained, this isolation rate accounts amongst the lowest reported hitherto throughout the world. Among the 30 NTM isolates that were available for culture, 27 (90.0%) have been identified to the species level. The most commonly encountered species was *Mycobacterium kansasii* (23.3%) subtype 1. Strikingly, all *M. kansasii* cases were male patients originating from Bizerte, an industrialized region particularly known for iron industry. The remaining NTM species were *M. fortuitum* (16.6%)*, M. novocastrense* (16.6%)*, M. chelonae* (10.0%), *M. gordonae* (6.6%), *M. gadium* (6.6%), *M. peregrinum* (3.3%), *M. porcinum* (3.3%), and *M. flavescens* (3.3%). There were no bacteria of the *M. avium* complex, the most frequently isolated NTM globally, and the main driver of the rise of NTM-lung diseases.

**Conclusions:**

This study uncovered an exceptional low prevalence of PNTM isolation among HIV-negative TB suspects in Northern Tunisia, suggesting a very low burden of NTM pulmonary disease. However, the frequent isolation of *M. kansasii* subtype 1, the most pathogenic subtype, particularly from the industrialized region of Bizerte, strongly suggests its effective involvement in a typical pulmonary disease.

**Supplementary information:**

**Supplementary information** accompanies this paper at 10.1186/s12879-019-4441-1.

## Background

Mycobacteria encompass a large taxonomic group distributed in various aquatic and terrestrial environments. These are acid-fast bacilli (AFB) belonging to the genus *Mycobacterium*. Unlike obligate pathogenic species, *Mycobacterium tuberculosis* complex (MTBC) and *M. leprae*, the vast majority of mycobacteria are environmental organisms that usually act as opportunistic pathogens, causing localized (skin and soft tissues, lymph nodes, bones, lungs) or disseminated infections [1, 2]. These opportunistic species are often collectively termed nontuberculous mycobacteria (NTM), but are also known as atypical mycobacteria, pathogenic environmental mycobacteria, or mycobacteria other than tuberculosis. NTM species are generally subdivided on the basis of growth into rapid and slow growing mycobacteria (RGM and SGM, respectively) [3]. Like MTBC members, pathogenicity in NTM is predominantly correlated with slow growth [4].

Given their ubiquitous presence in the environment, NTM can colonize humans, especially through the upper airways, and may cause a severe pulmonary disease in both immunocompromised and immunocompetent individuals [5, 6]. Pulmonary infections due to NTM represent an emerging issue, particularly in Western countries where NTM prevalence may surpass TB [6]. There were significant differences in NTM species distribution within and between continents, which was suggested to influence the frequency and manifestations of pulmonary NTM (PNTM) disease in each geographical location [7].

In Tunisia, the national TB program (NTP) that was implemented in 1959 has significantly contributed to the overall decrease in TB incidence. Indeed, in the time period 1975 to 2002, TB incidence decreased from 48.6 down to 18.9 per 100,000 population. Yet, in the last decade, TB incidence has registered a steady increase, reaching 34.0 per 100,000 population in 2017 [8]. However, the magnitude of PNTM infection remains totally unknown.

While the geographical distribution of NTM isolation has been described worldwide [7], including sub-Saharan Africa [9], there are no data reported hitherto regarding Tunisia. Here, based on culture results of respiratory samples forwarded to the Institut Pasteur de Tunis during the time period 2002–2016, we estimated the prevalence of PNTM isolation among pulmonary TB suspects, originating mainly from Northern Tunisia, and determined the identity of the involved species.

## Methods

### Pulmonary samples

**Fig. 1:**
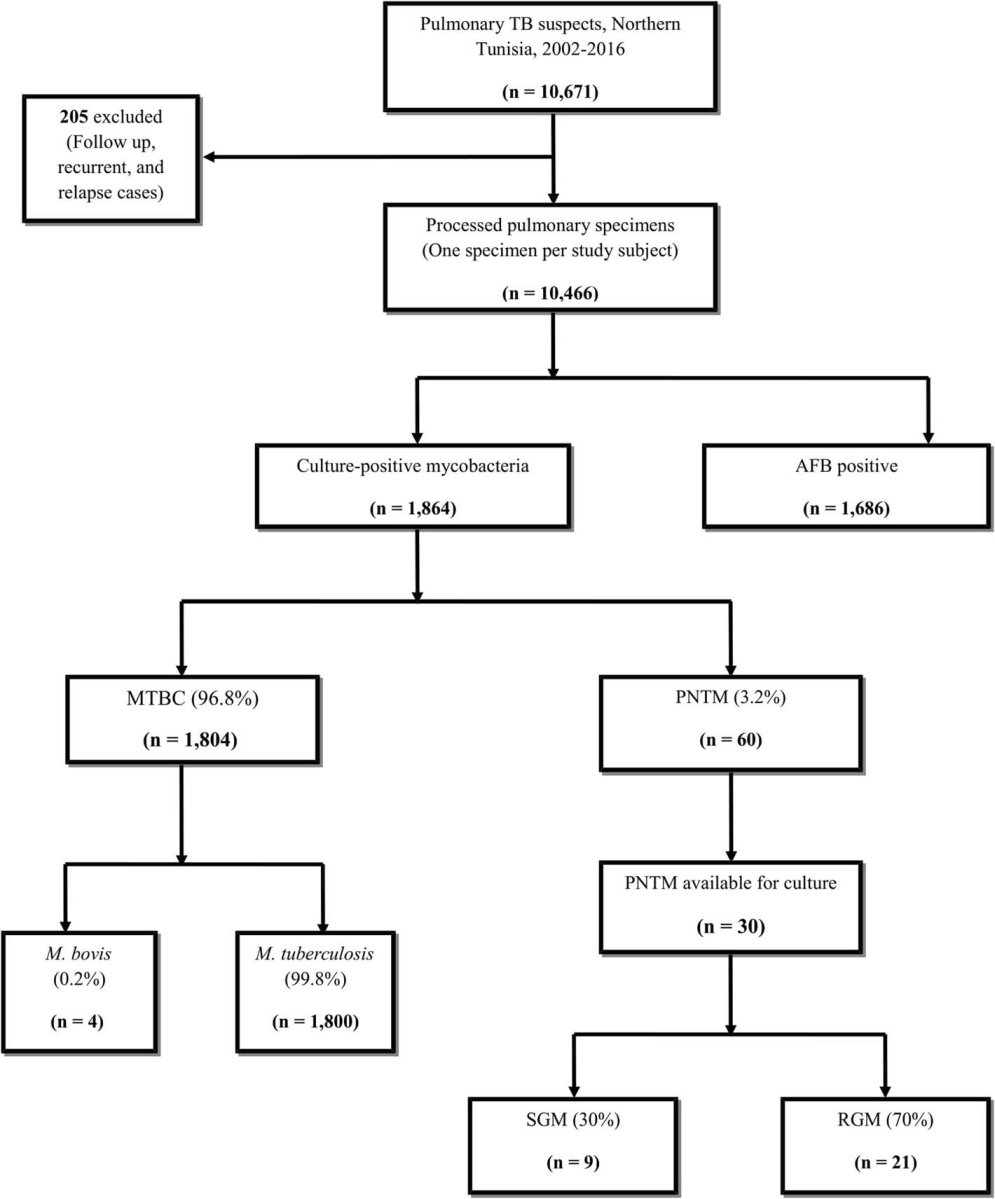
A Flow chart of specimen collection and mycobacterial isolation, Northern Tunisia, 2002–2016. AFB: acid-fast bacilli; MTBC: *Mycobacterium tuberculosis* complex; PNTM: pulmonary nontuberculous mycobacteria; SGM: slowly growing mycobacteria, RGM: rapidly growing mycobacteria

We carried out a retrospective review of mycobacterial cultures originating from Northern Tunisia (Tunis, Bizerte, and Zaghouan), which have been processed in the laboratory of mycobacteria of the Institut Pasteur de Tunis, Tunisia. From January 2002 to November 2016, a total of 10,671 pulmonary samples obtained from TB suspects have been processed (Fig. 1). After excluding follow up, recurrent, and relapse cases, the culture results of the remaining 10,466 specimens (7699 sputum, 2093 bronchial washes, 674 pleural fluids) were taken into account.

Except for a few cases, all processed pulmonary samples were from Tunisian patients who permanently resided in Tunisia. The vast majority of patients from Tunis lived in urban districts, whereas the majority of patients from the regions of Bizerte, and particularly those from Zaghouan, resided in rural areas. Virtually, all TB suspects from the latter two regions were admitted into their respective unique regional hospital, and hence, their forwarded specimens represented a full capture of the prevailing TB situation. By contrast, patients from Tunis, came from various healthcare sites from both the public and private sectors.

### Mycobacterial isolation and phenotypic characterization

Briefly, pulmonary specimens (sputum, pleural fluid, bronchial wash) were processed by the N-acetyl L cysteine-sodium hydroxide (NALC-NaOH) method, and inoculated into Löwenstein-Jensen (LJ) medium. Inoculated LJ slants were incubated at 37 °C for eight weeks and examined every week.

Differentiation between MTBC and NTM colonies were initially performed by four standard biochemical tests: niacin, nitrate, heat-resistant catalase test (HRCT) and para- nitro benzoic acid (PNB). The NTMs were confirmed by PCR targeting the *recA* intein, as previously described [10], and were further characterized phenotypically by morphological character and biochemical tests, i.e. colony morphology, growth rate, growth at various temperatures (25 °C, 37 °C and 44 °C), pigment production in dark (schotochromogen), pigment production on exposure of light (photochromogen), absence of pigment production (non-chromogen), semi-quantitative Catalase test (SQCT), Thiophene2-carboxylic acid hydrazide (TCH) susceptibility test, Tween 80 hydrolysis, Aryl sulphatase test (3 days and 14 days), and *β*-*Gal* activities.

### Data analysis

One isolate per patient was eligible for calculating PNTM isolation rate, which refers to the total number of PNTM divided by the total number of pulmonary specimens received in a year (annual rate) or during the study period (overall isolation rate). For patients with multiple PNTM isolations, we mostly took into consideration the very first isolate. PNTM isolation prevalence for the study period, or for a given year, was calculated as the total number of PNTM positive cultures in a particular region divided by the population estimate and expressed per 100,000 population. In this study, we used the 2014 estimates of the Tunisian population, the most recent official estimates available: Tunis (2,643,695 inhabitants), Bizerte (568,219 inhabitants), and Zaghouan (176,945 inhabitants).

### PCR amplification and nucleotide sequencing

From a few colonies cultivated on LJ slants, the DNA was extracted by thermolysis. For this purpose, the colonies are suspended in 100 μl of 1% EDTA / Triton Tris (TE / Triton) and inactivated at 80 °C for 30 min.

PCR amplifications and sequencing of the *rpoB*, 16S rRNA, *hsp65*, and s*odA* gene sequences were performed using the primer pairs listed in Additional file 1.

PCR reactions consisted of a15-min inactivation period followed by 35 cycles of 95 °C for 30s, 60  °C for 1 min (64 °C for 1 min for *rpoB*, 52 °C for 30 s for 16S rRNA) and 72 °C for 2 min, with a final extension step at 72 °C for 5–7 min.

PCR amplifications were performed in a 50-μl PCR mixture containing 5 μl 10x buffer (Qiagen, Courtaboeuf, France), 200 mM each dNTP, 1.5 mM MgCl_2_, 1.25 U HotStarTaq polymerase (Qiagen), 1 μl each primer (10 pM), 33 μl nuclease-free water and 5 μl DNA template. Negative controls consisting of PCR mixture without DNA template were included in each PCR run. The PCR products were visualized by gel electrophoresis, treated with *Shrimp Alkaline Phosphatase* (SAP) and *Exonuclease* I (ExoI) (Sigma-Aldrich,USA), and sequenced in both directions using the BigDye Terminator sequencing kit (Applied Biosystems, Villebon-sur-Yvette, France) with an ABI PRISM 3100 automatic sequencer (ABI, USA). The sequences generated in the present study were deposited to the GeneBank. Their accession numbers are provided in Additional file 2.

Nucleotide sequences were processed using BioEdit (BioEdit software, version 7.0.5), and aligned with the GenBank database (NCBI) using the Basic Local Alignment Search Tool (BLAST).

### Species identification

Species identification was primarily based on similarity rates with *rpoB* gene sequence of reference strains (http://www.bacterio.net/mycobacterium.html) [11]. Briefly, assignment of an isolate to a particular species was allowed if its *rpoB* sequence displayed at least 99.3% similarity to type strain for slow growers and 98.3% for rapidly growing mycobacteria. Isolates showing similarity rates below these thresholds but ≥94% were identified to complex level. Isolates displaying less than 94% similarity were referred to as *rpoB* unidentified *Mycobacterium* species (rUMS). With regard to 16S rRNA gene sequencing, identification was based on 100% matches.

Subtyping of *Mycobacterium kansasii* was performed by PCR-restriction fragment length polymorphism (PCR-RFLP) analysis of the *hsp65* gene as described previously [12].

### Phylogenetic analyses

Phylogenetic analyses were carried out with MEGA (Molecular Evolutionary Genetics Analysis, version 7.0) [13]. Phylogenetic tree analyses were performed using the neighbor-joining method based on the Kimura two-parameter model with 1000 bootstrap replicates. Nocardia nova was used as the outgroup.

## Results

**Fig. 2:**
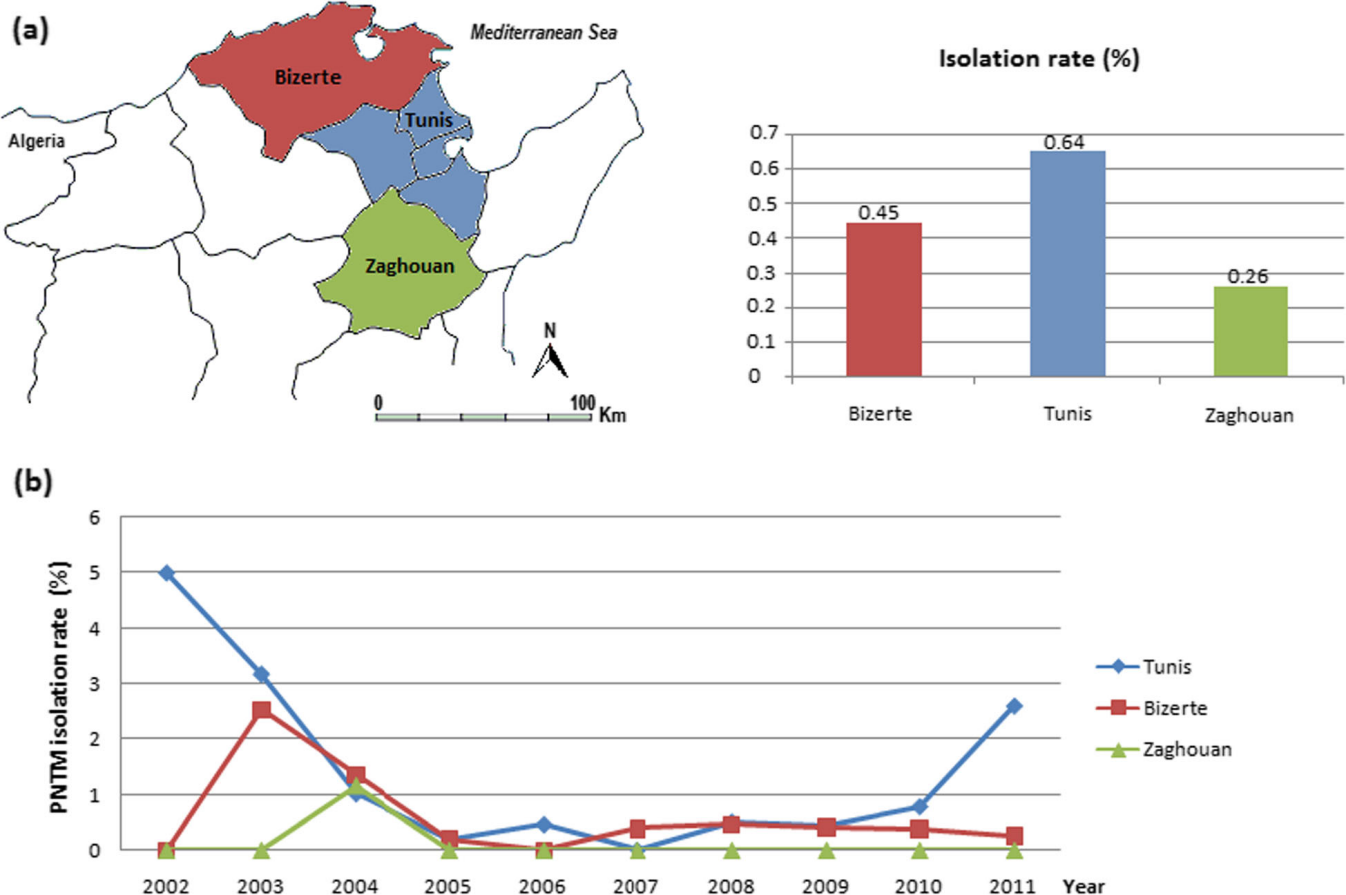
(**a**) Map of Northern Tunisia showing PNTM isolation rates by region. The map was generated using Base map from https://d-maps.com; (**b**) Annual PNTM isolation rates (2002–2011) by region

Of the 10,466 specimens that were processed in the laboratory of Mycobacteria of the Institut Pasteur de Tunis for presumptive clinical pulmonary TB, 1864 mycobacteria-positive cultures were obtained, 96.8% of which belonged to the *Mycobacterium tuberculosis* complex (MTBC) (Fig. 1). PNTM isolation occurred in 60 (3.2%) cases (54 isolates from sputum and 6 isolates from bronchial washes). All patients from whom PNTM isolates were obtained were HIV-negative, the majority of whom (*N* = 42, 70.0%) were male. The distribution of isolated PNTMs according to the geographical region was as follows: 41 (68.0%) from Tunis, 18 (30.0%) from Bizerte, and 1 (2.0%) from Zaghouan. The overall PNTM isolation rate was found to be 0.6% for the time period 2002–2016. PNTM isolation rates by region and/or per year are shown in Fig. 2a and Fig. 2b, respectively.

Since we ensured full coverage of the regions of Bizerte and Zaghouan during the time period 2002–2011, one can reliably estimate their respective mean annual prevalence of PNTM isolation, which were found to be 0.3/100,000 and 0.1/100,000, yielding an overall annual PNTM isolation prevalence of 0.2/100,000. It is worth noting that, during this time period, only one PNTM isolate was recovered from the 383 processed specimens from Zaghouan.

Initial classification of the cultured bacteria as NTM was based on their acid fastness and biochemical characteristics, and next confirmed by PCR targeting the *recA* intein (data not shown). Among the 30 cultured PNTM isolates, 9 (30.0%) were slow growers. Phenotypic characteristics of the isolated PNTMs are shown in Table 1.

**Table 1:** Epidemiologic and phenotypic features of NTM isolates recovered from Tunisian pulmonary TB suspects

Reference	Year of isolation	Origin	Specimen	Sex	Runyon Classification	BIOCHEMICAL TESTS								
						Niacin	Nit- Red	Urease	Catalase		Arylsulfatase		Tween 80	β-Gal
								3 days	22 °C	68 °C	3 days	14 days		
TNTM1	2002	Bizerte	Sputum	M	P/SGM	**–**	**+**	**+**	**+**	**+**	**–**	**+**	**+**	**–**
TNTM2	2009	Bizerte	Sputum	M	P/SGM	**–**	**+**	**+**	**+**	**+**	**–**	**+/−**	**+**	**–**
TNTM3	2009	Bizerte	Sputum	M	P/SGM	**–**	**+**	**+**	**+**	**+**	**–**	**+/−**	**+**	**–**
TNTM4	2010	Bizerte	Sputum	M	P/SGM	**–**	**+**	**+**	**+**	**+**	**–**	**+/−**	**+**	**–**
TNTM5	2008	Bizerte	Sputum	M	P/SGM	**–**	**+**	**+**	**+**	**+**	**–**	**+**	**+**	**–**
TNTM6	2002	Bizerte	Sputum	M	P/SGM	**–**	**+**	**+**	**+**	**+**	**–**	**+**	**+**	**–**
TNTM7	2007	Bizerte	Sputum	M	P/SGM	**–**	**+**	**+**	**+**	**+**	**–**	**+**	**+**	**–**
TNTM8	2000	Tunis	Sputum	M	S/SGM	**–**	**–**	**–**	**+**	**+**	**–**	**+**	**+**	**–**
TNTM9	2004	Tunis	Sputum	M	S/SGM	**–**	**–**	**–**	**+**	**+**	**–**	**+**	**+**	**–**
TNTM10	2003	Tunis	Sputum	F	N/RGM	**–**	**+**	**+**	**+**	**+**	**+**	**+**	**–**	**–**
TNTM11	2011	Tunis	Sputum	M	N/RGM	**–**	**+**	**+**	**+**	**+**	**+**	**+**	**–**	**–**
TNTM12	2015	Tunis	Sputum	F	N/RGM	**–**	**+**	**+**	**+**	**+**	**+**	**+**	**–**	**–**
TNTM13	2011	Tunis	Sputum	F	N/RGM	**–**	**+**	**+**	**+**	**+**	**+**	**+**	**–**	**–**
TNTM14	2005	Tunis	Sputum	M	N/RGM	**–**	**+**	**+**	**+**	**+**	**+**	**+**	**–**	**–**
TNTM15	2004	Tunis	Sputum	M	N/RGM	**–**	**+**	**+**	**+**	**+**	**+**	**+**	**–**	**–**
TNTM16	2008	Bizerte	Sputum	M	N/RGM	**–**	**–**	**+**	**+**	**+**	**+**	**+**	**–**	**–**
TNTM17	2003	Bizerte	Sputum	M	P/RGM	**–**	**+**	**+**	**+**	**+**	**+**	**+**	**–**	**–**
TNTM18	2008	Tunis	Sputum	M	P/RGM	**–**	**+**	**–**	**+**	**+**	**+**	**+**	**–**	**–**
TNTM19	2008	Tunis	Sputum	F	P/RGM	**–**	**+**	**–**	**+**	**+**	**+**	**+**	**–**	**–**
TNTM20	2009	Tunis	Sputum	M	P/RGM	**–**	**+**	**+**	**+**	**+**	**+**	**+**	**–**	**–**
TNTM21	2004	Tunis	Sputum	F	P/RGM	**–**	**+**	**+**	**+**	**+**	**+**	**+**	**–**	**–**
TNTM22	2016	Tunis	Sputum	M	S/RGM	**–**	**+**	**+**	**+**	**+**	**+**	**+**	**+**	**–**
TNTM23	2003	Bizerte	Sputum	M	S/RGM	**–**	**+**	**+**	**+**	**+**	**–**	**–**	**–**	**–**
TNTM24	2003	Tunis	Sputum	M	S/RGM	**–**	**+**	**+**	**+**	**+**	**–**	**–**	**–**	**–**
TNTM25	2009	Tunis	Sputum	M	N/RGM	**–**	**–**	**+**	**+**	**+**	**+**	**+**	**–**	**–**
TNTM26	2010	Tunis	Sputum	F	N/RGM	**–**	**–**	**+**	**+**	**+**	**+**	**+**	**–**	**–**
TNTM27	2009	Tunis	Sputum	M	N/RGM	**–**	**–**	**+**	**+**	**+**	**+**	**+**	**–**	**–**
TNTM28	2010	Tunis	Sputum	M	N/RGM	**–**	**+**	**+**	**+**	**+**	**+**	**+**	**–**	**–**
TNTM29	2004	Tunis	Sputum	F	S/RGM	**–**	**–**	**+**	**+**	**+**	**+**	**+**	**–**	**–**
TNTM30	2008	Tunis	Sputum	M	S/RGM	**–**	**+**	**+**	**+**	**+**	**+**	**+**	**–**	**–**

*Abbreviations*: *M* Male, *F* Female, *P* Photochromogen, *S* Scotochromogen, *N* Non chromogen, *SGM* Slowly Growing Mycobacteria, *RGM* Rapidly Growing Mycobacteria, *Niacin* Niacin production, *NIT red* Nitrate reduction, *Urease* Urease activity after 3 days, *Tween 80* Tween 80 hydrolysis, *β-Gal* β-Galactosidase activity

All PNTM isolates were subjected to *rpoB*, 16S rRNA, *hsp65* and *sodA* gene in an attempt to unambiguously assign a species to each of them. Based on BLAST analysis using *rpoB* sequence data, following the previously adopted classification criteria [11], we could assign all but 2 isolates (90.0% classification rate) to one of the following species/complex: *M. kansasii, M. gordonae, M. fortuitum/M. fortuitum complex, M. Novocastrense, M. peregrinum, M. porcinum, M. flavescens, M. gadium, and M. chelonae* (Table 2). In comparison, 16S rRNA sequencing allowed identifying 25 isolates to the species level (83.3%) (Table 2).

**Table 2:** Species assignments of Tunisian PNTM isolates according to % similarity with *rpoB*, 16S rRNA, *hsp65*, and *sodA* gene sequences

Reference	Species identification based on % similarity			
	*rpoB*	16S rRNA gene	*hsp65*	*sodA*
TNTM1	*M. kansasii* (100%)	*M. kansasii* (100%)	*M. kansasii* (100%)	*M. kansasii* (100%)
TNTM2	*M. kansasii* (100%)	*M. kansasii* (100%)	*M. kansasii* (100%)	*M. kansasii* (100%)
TNTM3	*M. kansasii* (100%)	*M. kansasii* (100%)	*M. kansasii* (100%)	*M. kansasii* (100%)
TNTM4	*M. kansasii* (100%)	*M. kansasii* (100%)	*M. kansasii* (100%)	*M. kansasii* (100%)
TNTM5	*M. kansasii* (100%)	*M. kansasii* (100%)	*M. kansasii* (100%)	*M. kansasii* (100%)
TNTM6	*M. kansasii* (100%)	*M. kansasii* (100%)	*M. kansasii* (100%)	*M. kansasii* (100%)
TNTM7	*M. kansasii* (100%)	*M. kansasii* (100%)	*M. kansasii* (100%)	*M. kansasii* (100%)
TNTM8	*M. gordonae* (100%)	*M. gordonae* (100%)	*M. gordonae* (97.64%)	*M. gordonae* (99.76%)
TNTM9	*M. gordonae* (100%)	*M. gordonae* (100%)	*M. gordonae* (100%)	*M. gordonae* (100%)
TNTM10	*M. fortuitum* (100%)	*M. fortuitum* (100%)	*M. fortuitum* (100%)	*M.fortuitum* (99.76%)
TNTM11	*M. fortuitum* (100%)	*M. fortuitum* (100%)	*M. fortuitum* (100%)	*M. fortuitum* (99.76%)
TNTM12	*M. fortuitum* (100%)	*M. fortuitum* (100%)	*M. fortuitum* (100%)	*M. fortuitum* (99.76%)
TNTM13	*M.fortuitum* (100%)	*M. fortuitum* (100%)	*M. fortuitum* (100%)	*M. fortuitum* (99.76%
TNTM14	*M.fortuitum* (100%)	*M. fortuitum* (100%)	*M. fortuitum* 100%	*M. fortuitum* (99.76%)
TNTM15	*M. peregrinum* (100%)	*M. peregrinum* (100%)	*M.peregrinum* (100%)	*M. peregrinum* (100%)
TNTM16	*M. porcinum* (100%)	*M.porcinum* (100%)	*M. porcinum* (99.71%)	*M. porcinum* (100%)
TNTM17	*M. novocastrense* (99.99%)	*M. novocastrense* (100%)	*M. novocastrense* (100%)	*M. novocastrense* (100%)
TNTM18	*M. novocastrense* (100%)	*M. novocastrense* (99.73%)	*M. novocastrense* (100%)	*M. novocastrense* (100%)
TNTM19	*M. novocastrense* (100%)	*M. novocastrense* (99.73%)	*M. novocastrense* (100%)	*M. novocastrense* (100%)
TNTM20	*M. novocastrense* (99.85%)	*M. novocastrense* (100%)	*M .novocastrense* (100%)	*M. novocastrense* (100%)
TNTM21	*M. novocastrense* (99.85%)	*M. novocastrense* (99.73%)	*M. novocastrense* (100%)	*M. novocastrense* (100%)
TNTM22	*M. flavescens* (99.98%)	*M. flavescens* (99.74%)	*M. flavescens* (99.41%)	*M. flavescens* (100%)
TNTM23	*M. gadium* (100%)	*M. gadium* (100%)	*M. gadium* (100%)	*M. gadium* (100%)
TNTM24	*M. gadium* (98.45%)	*M. gadium* (100%)	*M. gadium* (98.82%)	*M. gadium* (100%)
TNTM25	*M. chelonae* (100%)	*M. chelonae* (100%)	*M. chelonae* 100%	*M. chelonae* (100%)
TNTM26	*M. chelonae* (100%)	*M. chelonae* (100%)	*M. chelonae* (100%)	*M. chelonae* (100%)
TNTM27	*M. chelonae* (100%)	*M. chelonae* (100%)	*M. chelonae* (100%)	*M. chelonae* (100%)
TNTM28	*M. peregrinum* (94.06%)	*M. fortuitum complex* (100%)	*M. sp H12O-99,072* (96.76%)	*M. fortuitum* (92.95%)
TNTM29	rUMS (90.03%)	*M. sp GR2001–270* (100%)	*M. sp* GR 2001–270 (97.64%)	*M. aurum* (90.10%)
TNTM30	rUMS (92.16%)	*M. sp G1368* (99.86%)	*M. sp* G1368 (96%)	*M. abscessus* (97.80%)

Among the 9 SGM isolates, seven (77.7%) were identified as *M. kansasii*, making up 23.3% (7/30) of all PNTMs characterized in this study. All *M. kansasii* isolates were confirmed by *16S rRNA, hsp65*, and *sodA* typing, since they displayed 100% similarity to the type strain, and all proved to be of subtype 1 (data not shown). The remaining two SGM isolates were identified as *M. gordonae* (100% similarity to type strain for both *rpoB* and *hsp65* gene sequences)*,* which is consistent with their phenotypic characteristics.

The most commonly encountered species amongst the characterized RGM isolates (*N* = 21) were *M. fortuitum* (*N* = 5, 23.8%)*, M. novocastrense* (N = 5, 23.8%)*, and M. chelonae* (*N* = 3, 14.3%), (Table 2). Indeed, these RGM isolates showed *rpoB* similarity rates ≥98.3% with their respective type strains. All *M. fortuitum* isolates were confirmed by both 16S rRNA and *hsp65* sequencing (100% similarity to type strain) (Table 2). One isolate, TNTM28, was considered as a member of the *M. fortuitum* complex based on 16S rRNA 100% similarity with all *M. fortuitum* species. It remains to be seen whether it represents a new species within this complex. Of the 5 *M. novocastrense* isolates, only two could be confirmed by 16S rRNA sequencing (100% similarity to type strain). All *M. chelonae* isolates displayed 100% similarity to type strain at their *hsp65* gene sequence. The remaining RGM isolates that were identified to the species level by *rpoB* sequencing are *M. gadium* (*N* = 2, 9.5%), *M. peregrinum* (*N* = 1, 4.8%), *M. porcinum* (N = 1, 4.8%), and *M. flavescens* (N = 1, 4.8%).

**Fig. 3:**
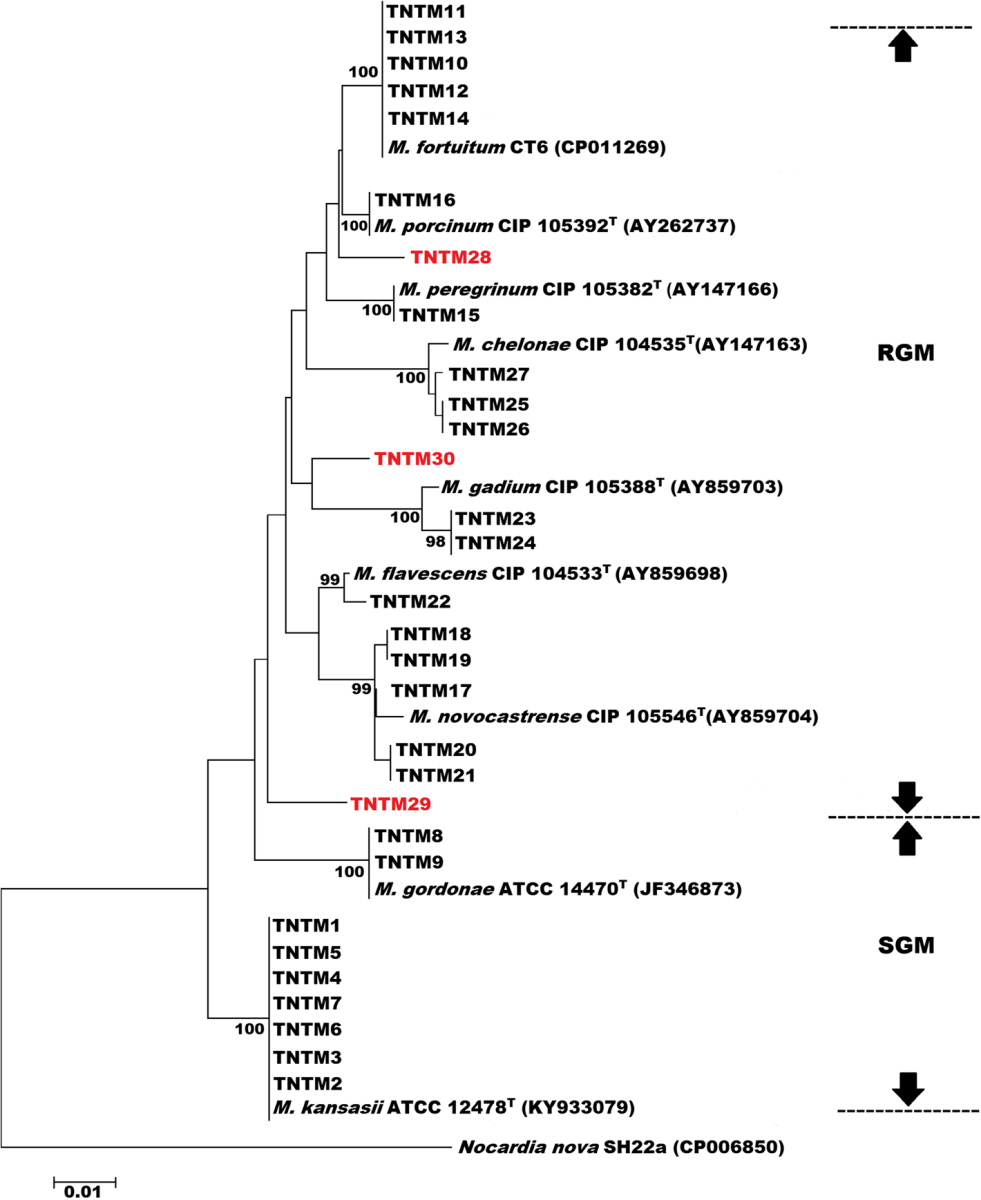
Phylogenetic tree based on *rpoB* gene using NJ method with Kimura 2-parameter distance correction model. The significance of branches (when > 50) is indicated by bootstrap values calculated on 1000 replicates. Bar, 1 substitution per 100 amino acid residues. PNTMs that could not be identified to the species level are highlighted in red

**Fig. 4:**
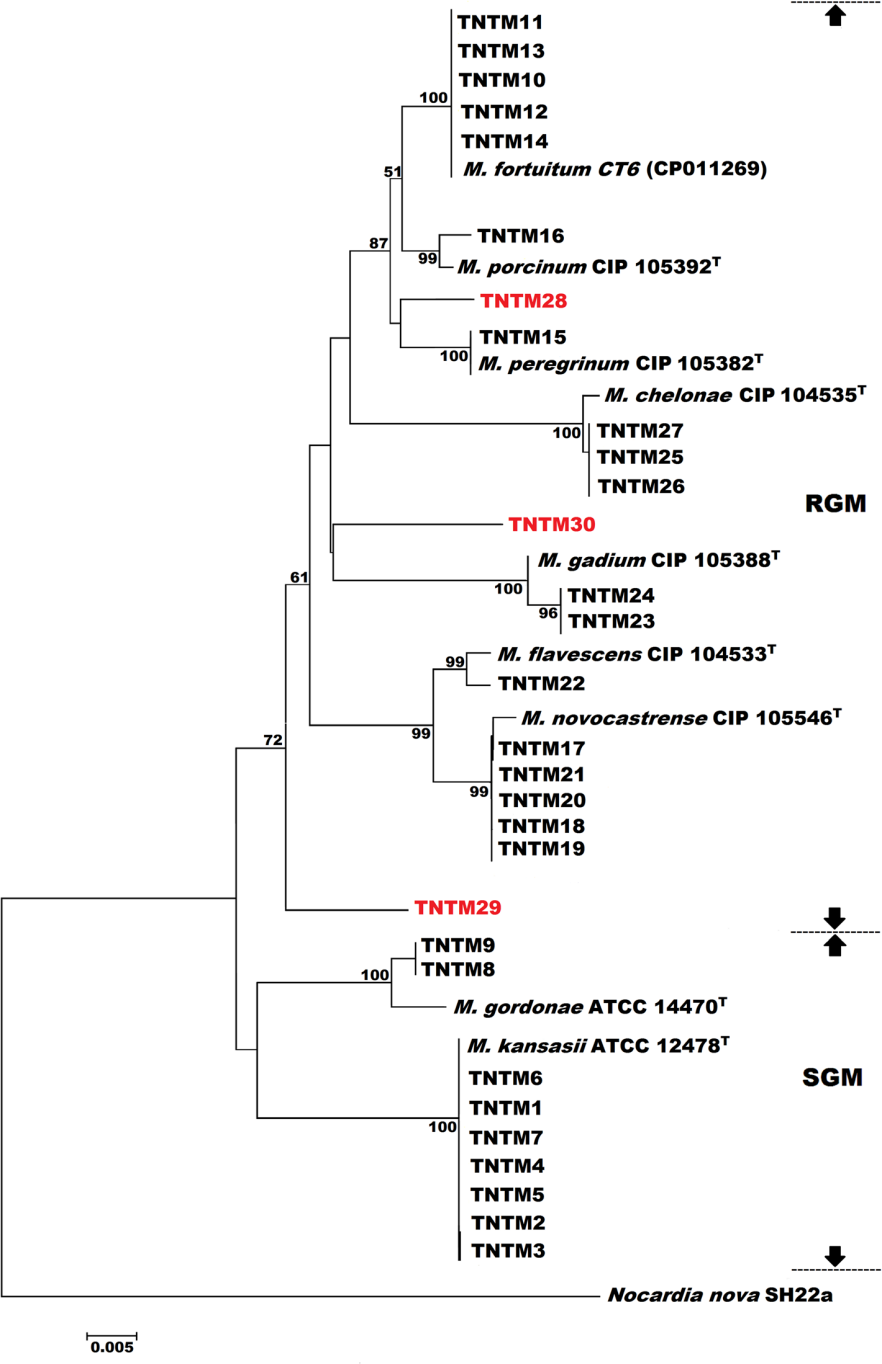
Phylogenetic tree based on concatenated *rpoB*, 16S rRNA, *hsp65* and *sodA* gene sequences using NJ method with Kimura 2-parameter distance model. The significance of branches (when > 50) is indicated by bootstrap values calculated on 1000 replicates. Bar, 5 substitutions per 100 amino acid residues. PNTMs that could not be identified to the species level are highlighted in red

Phylogenetic analyses using *rpoB* (Fig. 3), 16S rRNA (Additional file 3), and the *rpoB*-16S rRNA-*hsp65*-*sodA* concatenated sequence (Fig. 4) further confirmed the phylogenetic status of the 28 identified isolates.

Two RGM isolates could not be assigned a species or a complex, as they displayed less than 94% similarity to any known reference strain based on their *rpoB* sequence (Table 2). Furthermore, none of the two isolates showed 100% similarity to any known reference strain based on their 16S rRNA, *hsp65*, or *sodA* gene sequences. The phylogenetic position of these two smooth chromogenic RGM isolates, TNTM29 and TNTM30, varied from one tree to another with low bootstrapping values. In all phylogenetic trees (Fig. 3, Additional file 3, and Fig. 4), TNTM29 and TNTM30 are the unique representative of their respective branch, generally occupying ancestral positions. Hence, the latter two isolates warrant further investigations as they may represent novel mycobacterial species.

## Discussion

This study was primarily conducted to provide an insight into the extent of NTM involvement in respiratory tract infections in Northern Tunisia, and to uncover the identity of the isolated species. To our knowledge, this is the first study addressing this issue in North Africa. Our study covered a long time period of ~ 15 years and encompassed a large geographic area of Northern Tunisia, including the greatest Tunis (the capital and surrounding areas), as well as the regions of Bizerteand Zaghouan. Therefore, we believe it best captured the rates of NTM isolation among pulmonary TB suspects in the Tunisian population.

The overall PNTM isolation rate of 0.6% in Northern Tunisia was comparable to the rate of 1.0% previously reported among HIV-negative patients from Portugal [14]. However, these rates are low compared to the mean rate of 7.5% estimated for nine sub-Saharan African countries [9], or other regions where the prevalence of NTM isolation from pulmonary samples was ~ 5 to 16 times higher; being 2.6% in China (Shanghai) [15], 3.2% in Iran [16], and 4.1 to 8.0% in Brazil [17, 18]. Hence, NTM isolation from pulmonary samples in Northern Tunisia occurred very rarely with an overall annual prevalence of 0.17/100,000. This rate is significantly low compared to Europe (2.9–7.0/100,000) [19–23], as well as South and North America (5.3–22.2/100,000) [24–28]. The low prevalence of NTM isolation in Northern Tunisia might stem from the fact that HIV infection is of very low prevalence (~ 0.1%) in this country [29]. Another explanation would be that for decades, the Tunisian population has been massively vaccinated with BCG, at birth and at entry to school, a practice that could have contributed to reduce NTM infection rates [30]. Furthermore, we believe that the use of the classical decontamination protocol coupled to culture in Löwenstein-Jensen medium might not be optimal for NTM isolation [31, 32].

In this retrospective review we have not been able to extract reliable and complete clinical data in order to be able to estimate the real prevalence of NTM pulmonary disease. However, since it has been estimated that roughly 50%, or even more less, of individuals whose respiratory cultures are positive for NTM fulfilled clinical criteria for NTM lung disease [5], one can rightfully argue that the overall prevalence of active respiratory infections due to NTM in Northern Tunisia is likely to be very low. Yet, this finding prompts a nationwide prospective study in order to determine the real prevalence of NTM respiratory disease in Tunisia.

Undoubtedly, the most common organism associated with pulmonary disease worldwide is by far the *Mycobacterium avium* complex (MAC), which includes at least 12 subspecies [33]. Pulmonary infection due to MAC was mainly associated with HIV infection [33]. The finding that no species belonging to MAC was isolated from our collection of respiratory samples is consistent with the very low prevalence (~ 0.1%) of HIV infection in Tunisia [29]. However, pulmonary disease due to MAC could also occur among HIV-negative patients. Hence, the rarity of isolation of MAC species from Tunisian patients might also reflect a specific susceptibility pattern of the study population. In this respect, it is worth mentioning that important geographic variations in the species of isolated PNTM were observed throughout the world [7].

By contrast, the slow growing *M. kansasii* was the most frequently isolated NTM from respiratory specimens in northern Tunisia (23.3%). *M. kansasii* is considered as one of the most pathogenic organism among all NTM species, and the presence of a single *M. kansasii* isolate in a sputum sample is generally interpreted as clinically significant [34]. This is particularly true in the United States and the United Kingdom where it was found that more than 70% of all patients with a respiratory isolate of *M. kansasii* had clinically relevant disease [35, 36]. Aerosols generated from water systems of habitation and industries are likely to be the major source of this NTM, whose isolation has also been related to mining activities [37]. Strikingly, all *M. kansasii* cases reported in this study are men originating from Bizerte, an industrialized region particularly known for iron industry. This observation warrants further investigation to understand the epidemiology of *M. kansasii* infection in this particular region and identify its potential reservoir. Furthermore, the finding that all *M. kansasii* clinical strains identified in Bizerte were of subtype 1, strongly suggests their involvement in a typical pulmonary disease [38].

## Conclusion

In summary, we provide the first insight into NTM pulmonary infection in Tunisia, reporting an exceptional low prevalence of PNTM isolation among HIV-negative TB suspects, thereby suggesting a very low burden of NTM pulmonary disease. However, the frequent isolation of *M. kansasii* subtype 1, the most pathogenic subtype, particularly from Bizerte, must alert the local health authorities therein in order to identify the source of infection.

## Supplementary information

**Additional file 1.** List of primers used in this study.

**Additional file 2.** Genbank sequence accession numbers.

**Additional file 3.** Phylogenetic tree based on 16S rRNA gene using NJ method with Kimura 2-parameter distance correction model. The significance of branches (when > 50) is indicated by bootstrap values calculated on 1000 replicates. Bar, 5 substitutions per 1000 amino acid residues. PNTMs that could not be identified to the species level are highlighted in red.

## Data Availability

The data and material included in this manuscript available from the corresponding author on reasonable request. The nucleotide data generated during the current study are available on GenBank nucleotide sequence database (https://www.ncbi.nlm.nih.gov/).

## Abbreviations

DNA: Deoxyribonucleic acid
MAC: *Mycobacterium avium* complex
MTBC: *Mycobacterium tuberculosis* complex
NTM: Non tuberculous mycobacteria
PCR: Polymerase chain reaction
PNTM: Pulmonary non tuberculous mycobacteria
RGM: Rapidly growing mycobacteria
SGM: Slowly growing mycobacteria
