# Supplementary material for: Nontuberculous mycobacteria isolated from specimens of pulmonary tuberculosis suspects, Northern Tunisia: 2002–2016
Source: BMC Infect Dis. 2019 Sep 18;19:819. doi: 10.1186/s12879-019-4441-1 (PMC6751674; doi:10.1186/s12879-019-4441-1)
Supplement: Supplementary file 1 — Additional file 1. List of primers used in this study. [file 12879_2019_4441_MOESM1_ESM.docx]

Additional file 1. List of primers used in this study.

| Primer | Sequence (5’-3’) | Orientation | Target gene | Amplicon size (bp) | Tm (°C) |
| --- | --- | --- | --- | --- | --- |
| TB11 | ACCAACGATGGTGTGTCCAT | Forward | hsp65 | 441 | 60 |
| TB12 | CTTGTCGAACCGCATACCCT | Reverse |  |  |  |
| MYCOF1 | TCCGATGAGGTGCTGGCAGA | Forward | rpoB | 764 | 64 |
| MYCOR2 | ACTTGATGGTCAACAGCTCC | Reverse |  |  |  |
| SODLGF | GAAGGAATCTCGTGGCTGAATAC | Forward | sodA | 541 | 60 |
| SODLGR | AGTCGGCCTTGACGTTCTTGTAC | Reverse |  |  |  |
| fDd1 | AGAGTTTGATCCTGGCTCAG | Forward | 16S rRNA | 1050 | 52 |
| rP2 | ACGGCTACCTTGTTACGACTT | Reverse |  |  |  |
