# Supplementary material for: Nontuberculous mycobacteria isolated from specimens of pulmonary tuberculosis suspects, Northern Tunisia: 2002–2016
Source: BMC Infect Dis. 2019 Sep 18;19:819. doi: 10.1186/s12879-019-4441-1 (PMC6751674; doi:10.1186/s12879-019-4441-1)
Supplement: Supplementary file 2 — Additional file 2. Genbank sequence accession numbers. [file 12879_2019_4441_MOESM2_ESM.docx]

**Genbank accession numbers**

***rpoB***

BankIt2210170 Seq1NTM1 MK751441

BankIt2210170 Seq2 MK751442

BankIt2210170 Seq3 MK751443

BankIt2210170 Seq4 MK751444

BankIt2210170 Seq5 MK751445

BankIt2210170 Seq6 MK751446

BankIt2210170 Seq7 MK751447

BankIt2210926 Seq8 MK762857

BankIt2210926 Seq9 MK762858

BankIt2210926 Seq10 MK762859

BankIt2210926 Seq11 MK762860

BankIt2210926 Seq12 MK762861

BankIt2210926 Seq13 MK762862

BankIt2210926 Seq14 MK762863

BankIt2211109 Seq15 MK762864

BankIt2211109 Seq16 MK762865

BankIt2211109 Seq17 MK762866

BankIt2211109 Seq18 MK762867

BankIt2211109 Seq19 MK762868

BankIt2211109 Seq20 MK762869

BankIt2211109 Seq21 MK762870

BankIt2211109 Seq22 MK762871

BankIt2211109 Seq23 MK762872

BankIt2211109 Seq24 MK762873

BankIt2211109 Seq25 MK762874

BankIt2211109 Seq26 MK762875

BankIt2211109 Seq27 MK762876

BankIt2211120 Seq28 MK762879

BankIt2211118 Seq29 MK762877

BankIt2211118 Seq30 MK762878

***hsp65***

BankIt2209477 Seq1 MK733611

BankIt2209477 Seq2 MK733612

BankIt2209477 Seq3 MK733613

BankIt2209477 Seq4 MK733614

BankIt2209477 Seq5 MK733615

BankIt2209477 Seq6 MK733616

BankIt2209477 Seq7 MK733617

BankIt2209072 Seq8 MK726278

BankIt2209072 Seq9 MK726279

BankIt2208577 Seq10 MK713929

BankIt2208577 Seq11 MK713930

BankIt2208577 Seq12 MK713931

BankIt2208577 Seq13 MK713932

BankIt2208577 Seq14 MK713933

BankIt2209073 Seq15 MK726280

BankIt2209622 Seq16 MK733618

BankIt2209079 Seq17 MK726281

BankIt2209079 Seq18 MK726282

BankIt2209079 Seq19 MK726283

BankIt2209079 Seq20 MK726284

BankIt2209079 Seq21 MK726285

BankIt2209092 Seq22 MK726291

BankIt2209091 Seq23 MK726289

BankIt2209091 Seq24 MK726290

BankIt2209081 Seq25 MK726286

BankIt2209081 Seq26 MK726287

BankIt2209081 Seq27 MK726288

BankIt2210139 Seq28 MK751438

BankIt2210139 Seq29 MK751439

BankIt2210139 Seq30 MK751440

***16S rRNA***

MK630253

MK630254

MK630255

MK630256

MK630257

MK630258

MK630259

MK630260

MK630261

MK630262

MK630263

MK630264

MK630265

MK630266

MK630267

MK630268

MK630269

MK630270

MK630271

MK630272

MK630273

MK630274

MK630275

MK630276

MK630277

MK630278

MK630279

MK630280

MK630281

MK630282

***sodA***

BankIt2211531 Seq1 MK765877

BankIt2211531 Seq2 MK765878

BankIt2211531 Seq3 MK765879

BankIt2211531 Seq4 MK765880

BankIt2211531 Seq5 MK765881

BankIt2211531 Seq6 MK765882

BankIt2211531 Seq7 MK765883

BankIt2211531 Seq8 MK765884

BankIt2211531 Seq9 MK765885

BankIt2211531 Seq10 MK765886

BankIt2211531 Seq11 MK765887

BankIt2211531 Seq12 MK765888

BankIt2211531 Seq13 MK765889

BankIt2211531 Seq14 MK765890

BankIt2211531 Seq15 MK765891

BankIt2211531 Seq16 MK765892

BankIt2211531 Seq17 MK765893

BankIt2211531 Seq18 MK765894

BankIt2211531 Seq19 MK765895

BankIt2211531 Seq20 MK765896

BankIt2211531 Seq21 MK765897

BankIt2211531 Seq22 MK765898

BankIt2211531 Seq23 MK765899

BankIt2211531 Seq24 MK765900

BankIt2211531 Seq25 MK765901

BankIt2211531 Seq26 MK765902

BankIt2211531 Seq27 MK765903

BankIt2211720 Seq28 MK778075

BankIt2211720 Seq29 MK778076

BankIt2211720 Seq30 MK778077
