# Supplementary figures and images for: Nontuberculous mycobacteria isolated from specimens of pulmonary tuberculosis suspects, Northern Tunisia: 2002–2016
Source: BMC Infect Dis. 2019 Sep 18;19:819. doi: 10.1186/s12879-019-4441-1 (PMC6751674; doi:10.1186/s12879-019-4441-1)

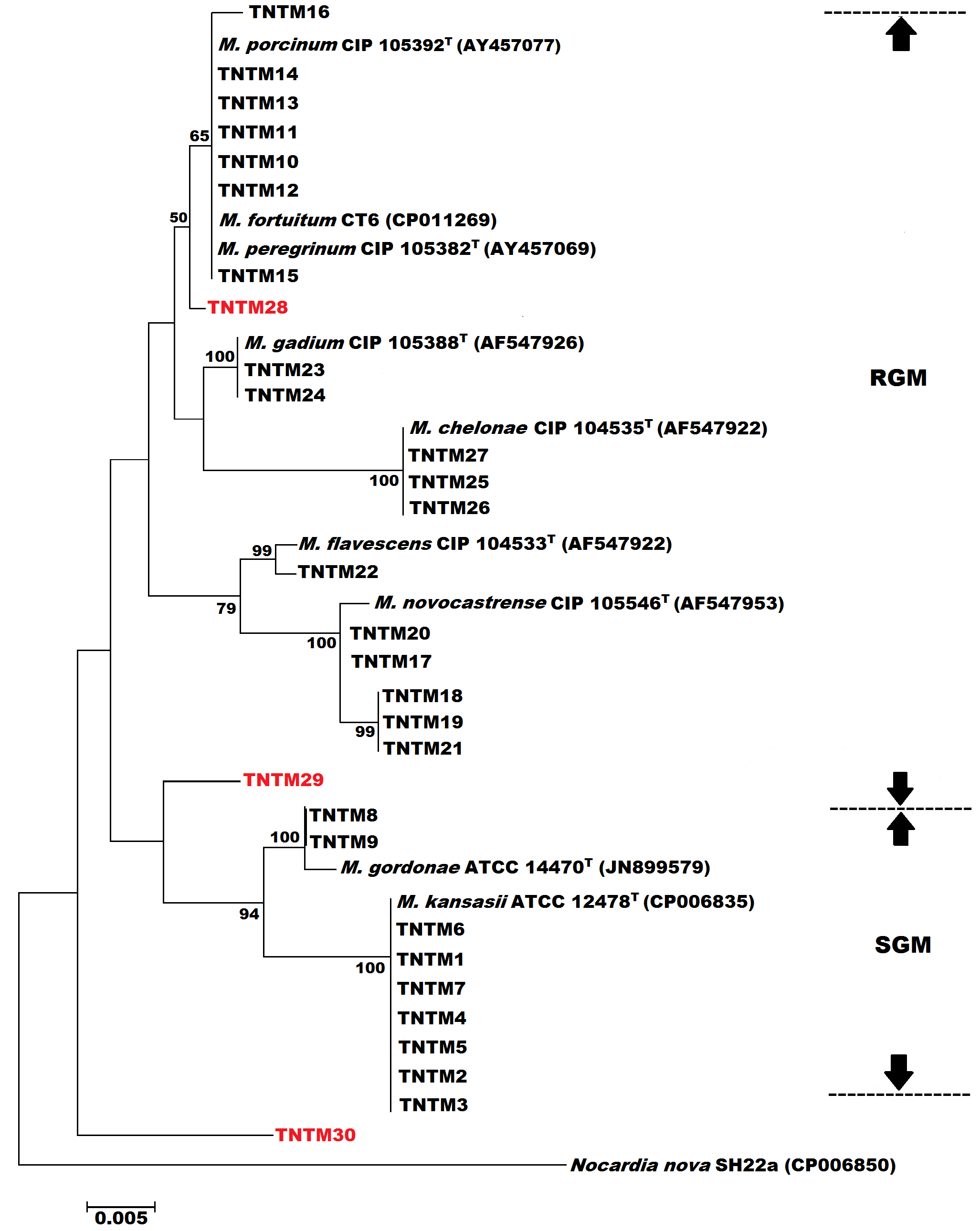

Supplement: Supplementary file 3 — Additional file 3. Phylogenetic tree based on 16S rRNA gene using NJ method with Kimura 2-parameter distance correction model. The significance of branches (when > 50) is indicated by bootstrap values calculated on 1000 replicates. Bar, 5 substitutions per 1000 amino acid residues. PNTMs that could not be identified to the species level are highlighted in red. [file 12879_2019_4441_MOESM3_ESM.tif]
